# Supplementary material for: A fln-2 mutation affects lethal pathology and lifespan in C. elegans
Source: Nat Commun. 2019 Nov 8;10:5087. doi: 10.1038/s41467-019-13062-z (PMC6841690; doi:10.1038/s41467-019-13062-z)
Supplement: Supplementary file 1 — Supplementary Information [file 41467_2019_13062_MOESM1_ESM.pdf]

Supplementary Information

A *fln-2* mutation affects lethal pathology and lifespan in *C. elegans*

Zhao et al.

Supplementary Figure 1. No phenotypic difference between N2H and N2M.
Supplementary Figure 2. Examples of pharynxes showing different stage of infection by OP50-RFP *E. coli*.
Supplementary Figure 3. Variant Discovery Mapping result of all chromosomes.
Supplementary Figure 4. *fln-2* isoforms and alleles.
Supplementary Figure 5. Effect of *fln-2* mutants on P.
Supplementary Figure 6. Effects of *fln-2* allele and FUDR on the lifespan effect of *geIs3 sir-2.1* over-expression strains.
Supplementary Figure 7. Effects of *pkIs1642 sir-2.1* low copy number over-expression on lifespan are FUDR dependent.
Supplementary Figure 8. The lifespan effect of *sir-2.1* null mutation.
Supplementary Table 1. Mortality deconvolution of N2H and N2M on proliferating and non-proliferating *E. coli*.
Supplementary Table 2. Mortality deconvolution of N2H-derived *fln-2(wu308)* Y800* mutant generated by CRISPR.
Supplementary Table 3. *fln-2* genotype of listed strains from *C. elegans* Expression Project (BC strains) and *C. elegans* Gene Knockout Project (FX, RB and VC strains).
Supplementary Table 4. Mortality deconvolution of the original *geIs3 sir-2.1* overexpression strains
Supplementary Table 5. Mortality deconvolution of *geIs3 sir-2.1* overexpression strains outcrossed with N2H
Supplementary Table 6. Mortality deconvolution of the original *geIs3 sir-2.1* overexpression strains without FUDR
Supplementary Table 7. Mortality deconvolution of the outcrossed *geIs3 sir-2.1* overexpression strains without FUDR
Supplementary Table 8. Mortality deconvolution of original and outcrossed *pkIs1642 sir-2.1* overexpression strains with and without FUDR
Supplementary Table 9. Mortality deconvolution of *sir-2.1(ok434)* mutant strains.
Supplementary Table 10. Mortality deconvolution of *daf-2* and *daf-2;daf-12* strains.
Supplementary Table 11. Mortality deconvolution of *eat-2(ad1116)* outcrossed strains.

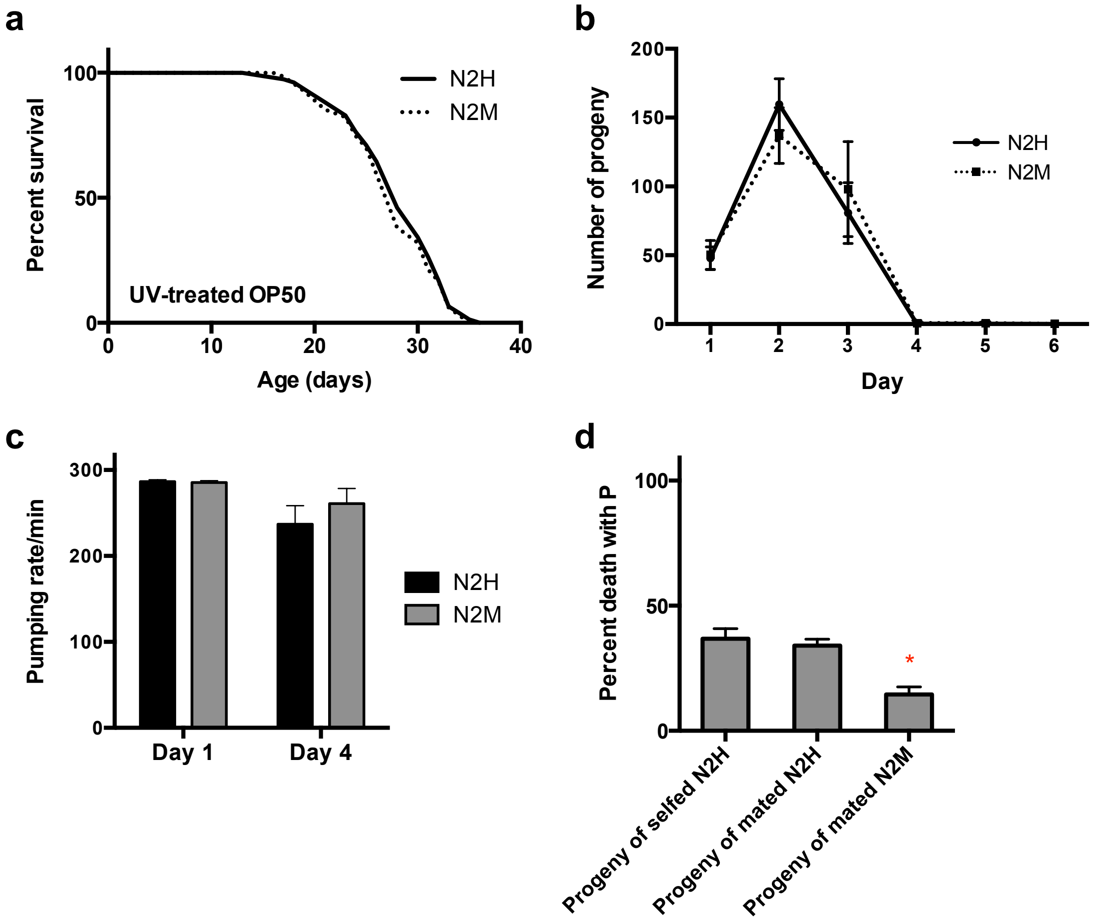

Supplementary Figure 1. No phenotypic difference between N2H and N2M.
(a) N2H and N2M showed no significant difference in lifespan on UV-treated bacteria (p=0.5672, log rank test). Graph shows pooled data from two trials. We noted a significant number of N2M animals dying with a ruptured vulva under this condition, however those animals were censored as in all lifespan experiments.
(b) N2H and N2M showed no significant difference in the timing and numbers of progeny. Error bar represents s.d, n = 20 for each strain. Source data are provided as a Source Data file.
(c) N2H and N2M showed no significant difference in pumping rate in early adulthood (day 1 and day 4) between N2H and N2M. Error bar represents s.e.m., n = 10 for each strain.
(d) Progeny of mated N2H are not resistant to pharyngeal infection. Error bar represents s.d. of two biological replicates.

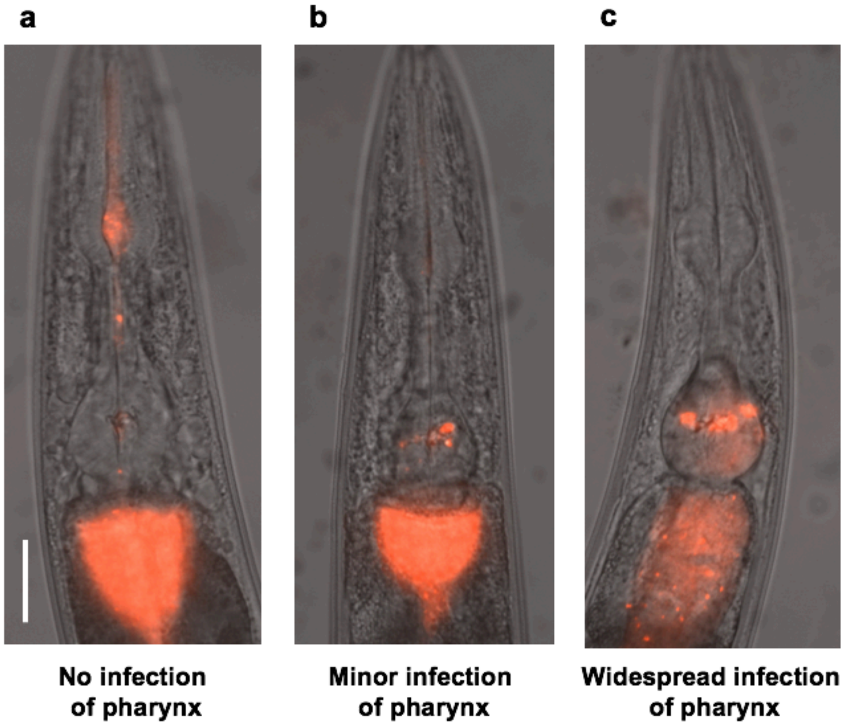

Supplementary Figure 2. Examples of pharynxes showing different stage of infection by OP50-RFP *E. coli*.

Worm showing (a) no bacterial invasion, (b) early or contained bacterial invasion, and (c) widespread infection in the posterior pharyngeal bulb. Examples shown are N2H worms on day 8 of adulthood.

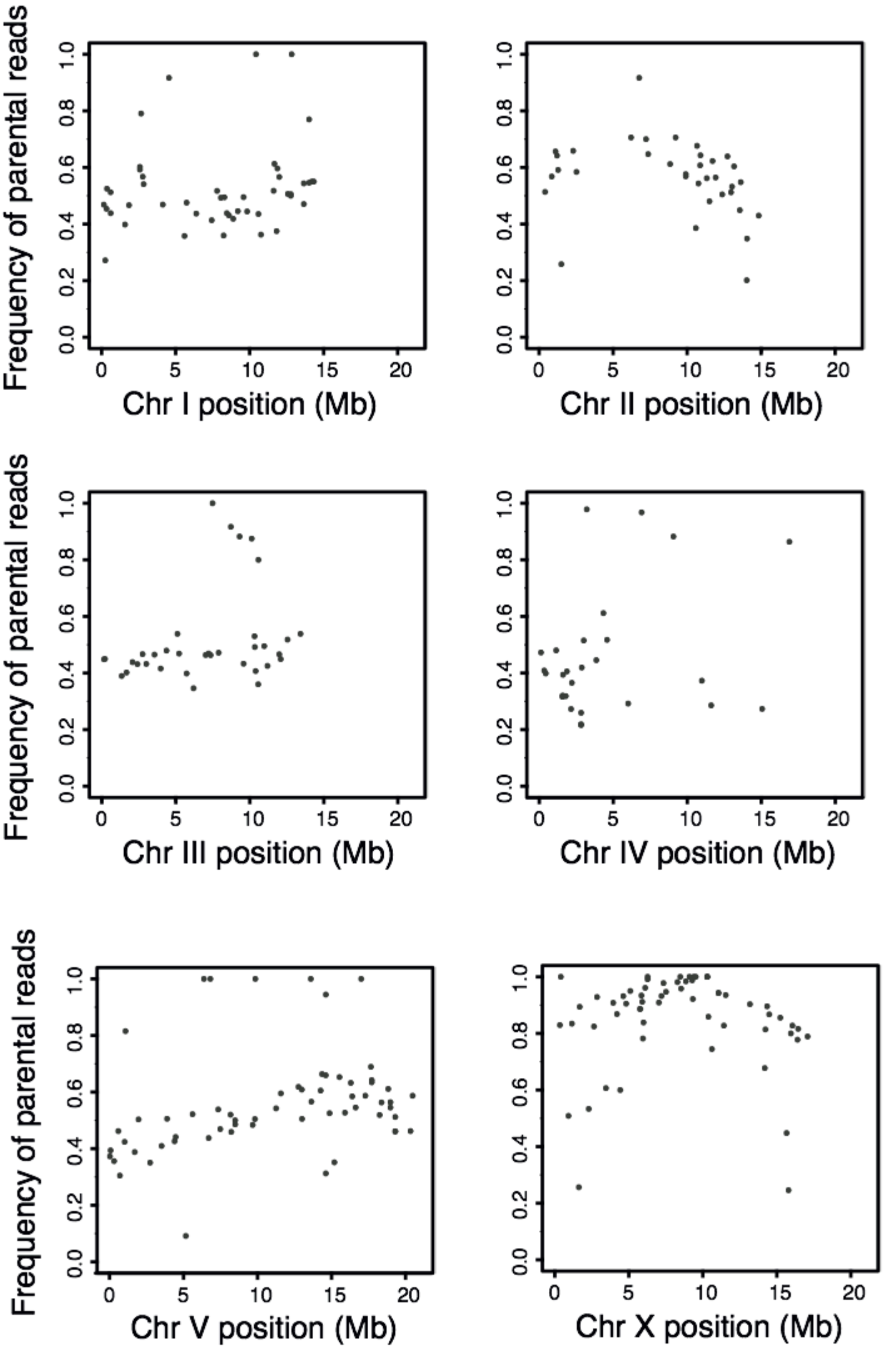

Supplementary Figure 3. Variant Discovery Mapping result of all chromosomes.

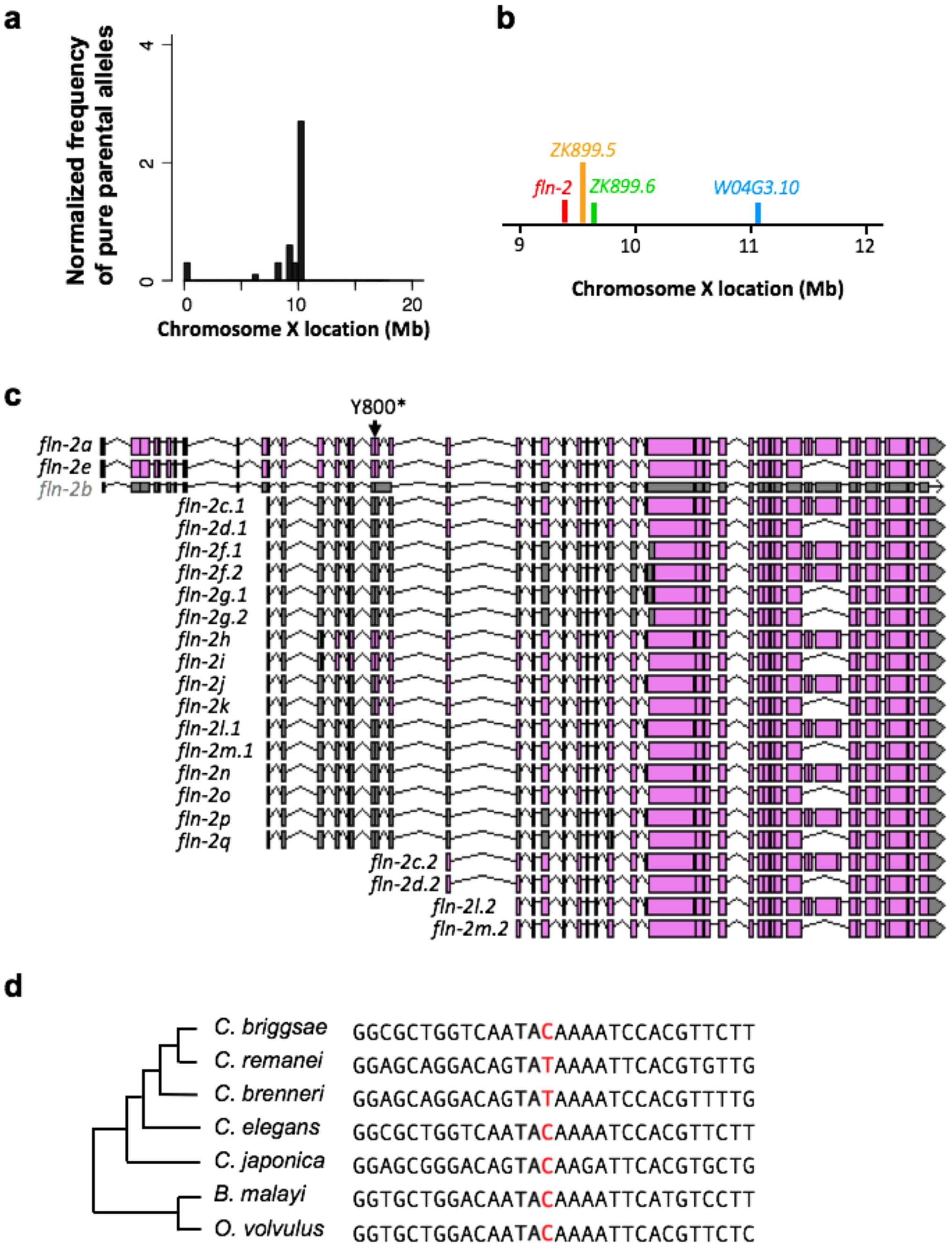

Supplementary Figure 4. *fln-2* isoforms and alleles.
(a) Normalized pure parental (N2M) allele frequency on chromosome X.
(b) Position of the missense mutations in the 9-12 MB region of chromosome X.
(c) Position of the nonsense mutation Y800* in *fln-2(ot611)* in relation to different *fln-2* isoforms, adapted from Wormbase.org.
(d) Sequence alignment of nematode *fln-2* orthologs.

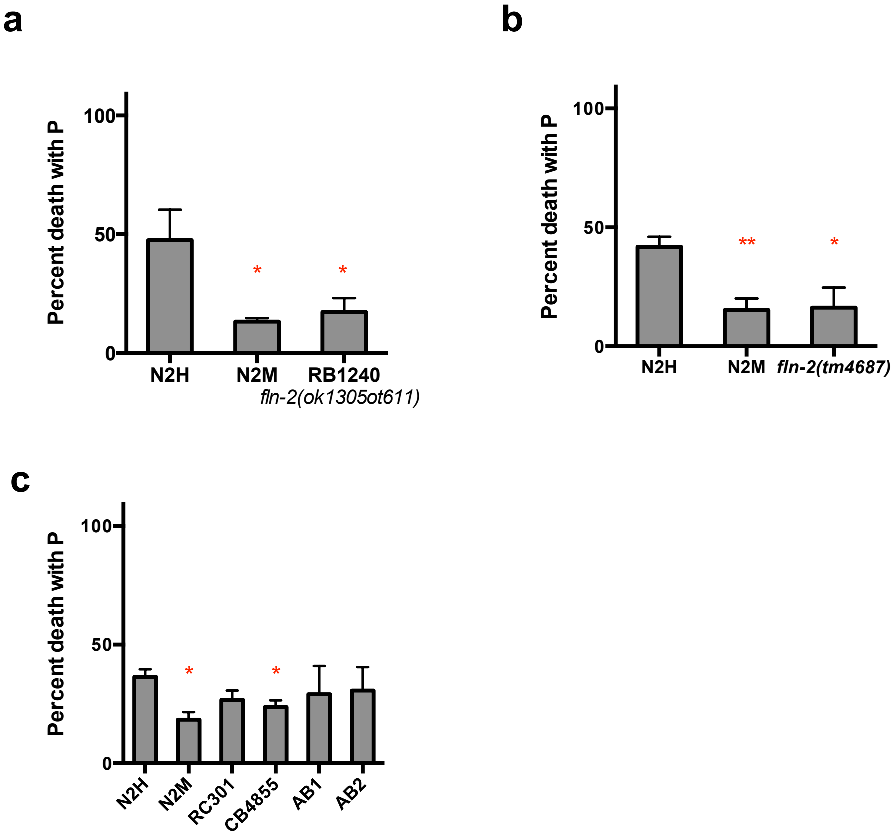

Supplementary Figure 5. Effect of *fln-2* mutants on P.
(a) RB1240 *fln-2(ok1305ot611)* is resistant to P death. Error bar represents s.d. of two trials. Source data are provided as a Source Data file.
(b) FX4687 *fln-2(tm4687)* is resistant to P death. Error bar represents s.d. of two trials.
(c) Frequency of P in *C. elegans* wild isolates. Error bar represents s.e.m. of three trials.

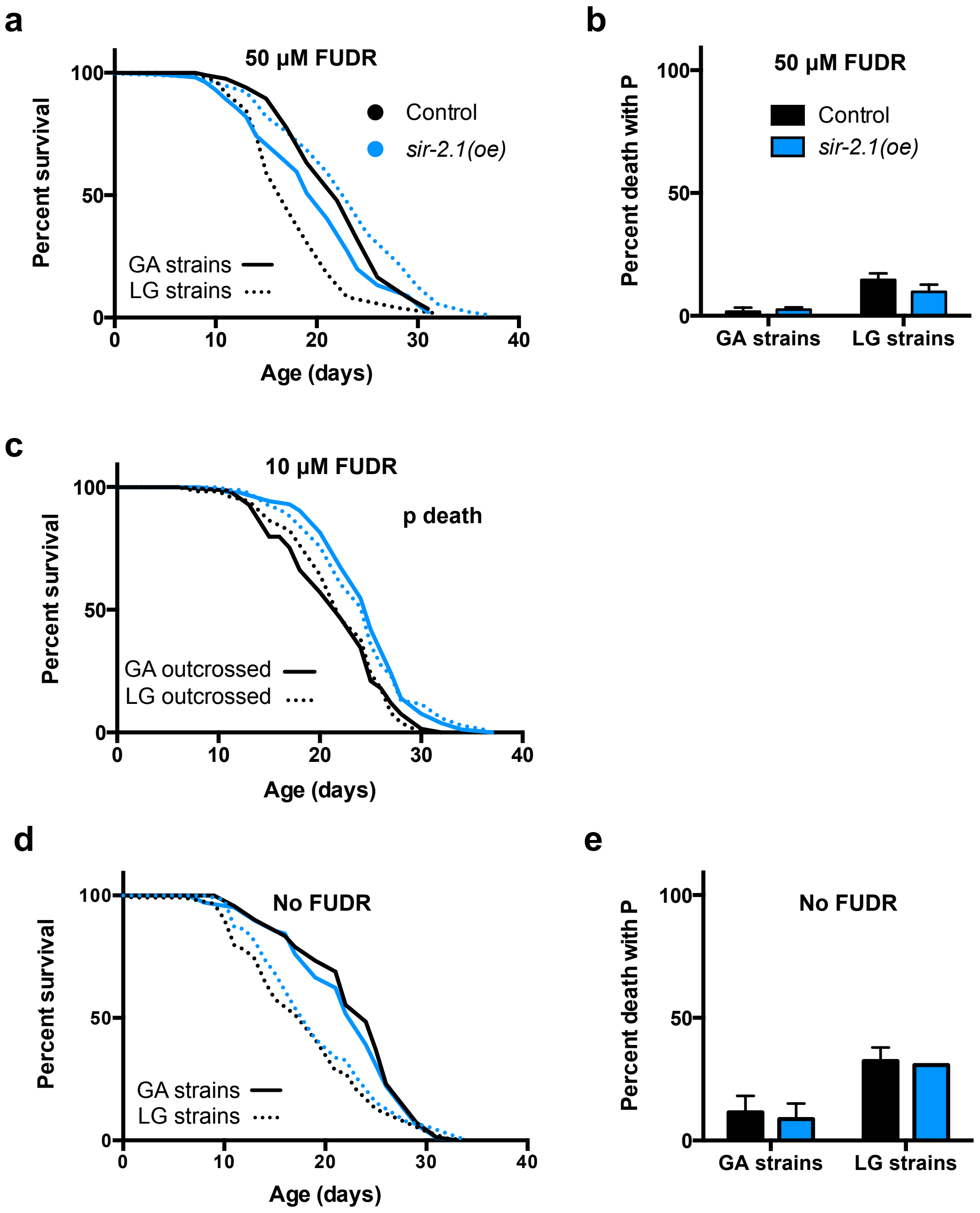

Supplementary Figure 6. Effects of *fln-2* allele and FUDR on the lifespan effect of *geIs3 sir-2.1* overexpression strains.

(a) Lifespan and (b) P death frequency of the original strains examined in the 2011 studies[1,2] in the presence of 50 µM FUDR. GA strains: GA707 *fln-2(ot611) wuEx166* control (black) and GA468 *geIs3 sir-2.1(oe); fln-2(ot611)* (blue). LG strains: LG398 *geIs101* control (black), LG394 *geIs3 sir-2.1(oe)* (blue).

(c) Lifespan of the p death subpopulation of the outcrossed strains in the presence of 10 µM FUDR, related to Fig. 3c.

(d) Lifespan and (e) P death frequency of *sir-2.1* over-expression and control strains as in (a), in the absence of FUDR. See Supplementary Table 6 for full statistics.

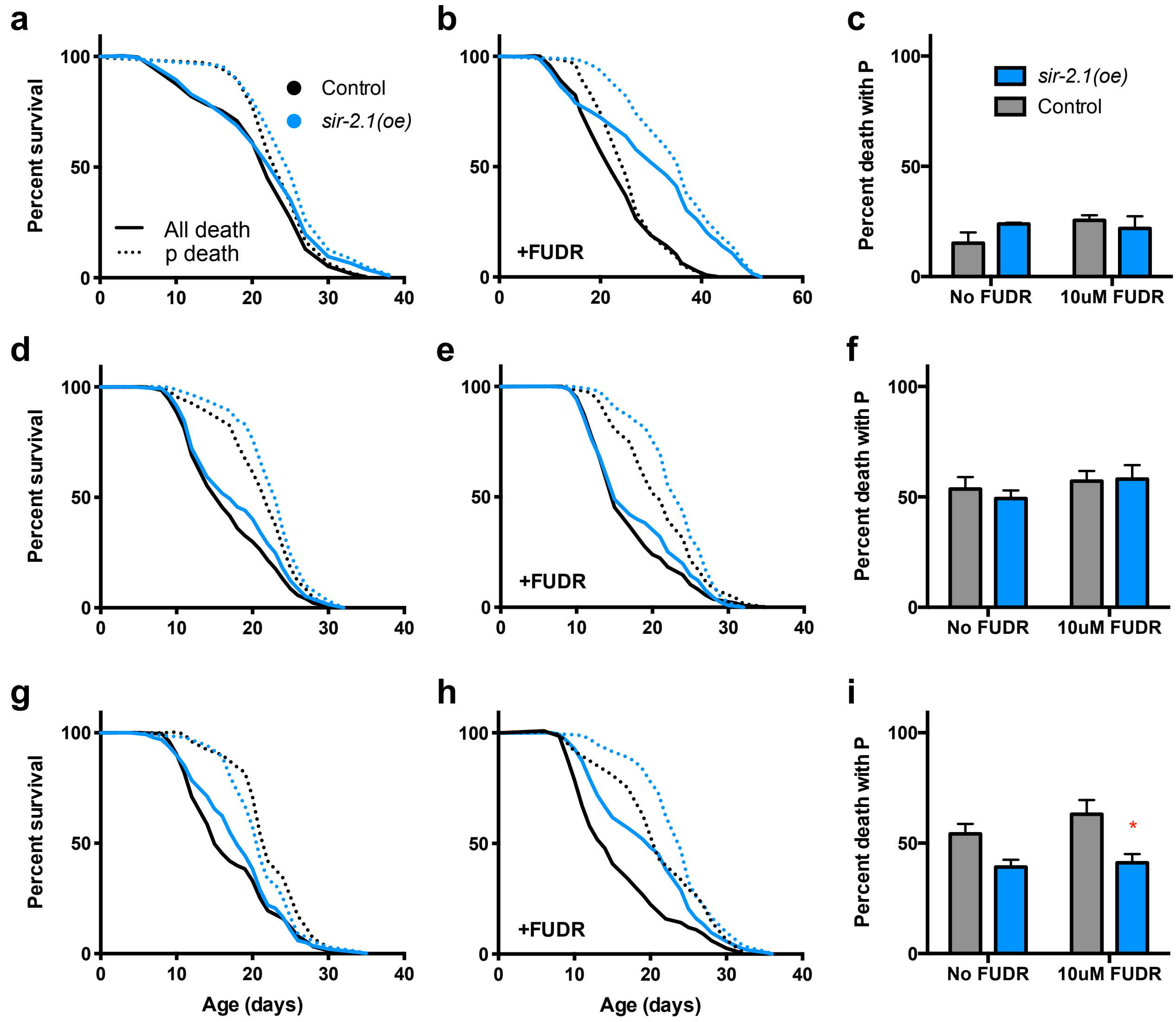

Supplementary Figure 7. Effects of *pkIs1642 sir-2.1* low copy number over-expression on lifespan are FUDR dependent.

(a-c) Strains used in the original study[3]: NL3909 *pkIs1642 sir-2.1(oe)* (blue) and NL3908 *pkIs1641* control (black). NL3909 was long lived in the presence (as seen previously[2]) but not the absence of FUDR (similar to another previous study[4]). P death frequency was abnormally low in both strains, although both were found to be *fln-2*(+), but wild-type P frequency was restored by backcrossing (see f, i), suggesting the presence of further genetic background variation reducing P frequency in NL3909 and NL3908. Here, instead, the FUDR-dependent extension of lifespan by *pkIs1642 sir-2.1(oe)* acts solely by an increase in p lifespan.

(d-f) Outcrossed (x2 with HT1593 *unc-119(ed3)*) strains described in a subsequent study: GA905 *pkIs1642 sir-2.1(oe)* (blue) and GA906 *pkIs1641* control (black)[2]. GA905 *sir-2(oe)* was not significantly longer lived than GA906 in the presence (as seen previously[2]) or absence of FUDR, nor was P frequency reduced in GA905.

(g-i) An additional set of independently outcrossed (x2) strains, SCS003 *pkIs1642 sir-2.1(oe)* (blue) and SCS004 *pkIs1641* control (black)[2] behaved similarly, but not identically to GA905 and GA906. In the absence of FUDR, SCS004 *sir-2.1(oe)* showed neither increased lifespan nor reduced P death frequency, consistent with a,c,d,f. However, in the presence of FUDR,

SCS003 *sir-2.1(oe)* showed increased overall lifespan, and reduced P death frequency. Thus, SCS003 *pkIs1642 sir-2.1(oe)* (low copy number) and GA468 *geIn3 sir-2.1(oe)* (high copy number) (Figure 3c,d) are similar in that in both strains *sir-2.1(oe)* is associated with an FUDR-dependent reduction of P frequency that contributes to increased lifespan. One possible reason for the different behavior of GA905 and SCS003 is that, despite backcrossing, the former retains background variation that blocks effects of *sir-2.1* and/or FUDR on pharyngeal infection.

Summed data from two (a-c) or three (d-i) trials; for survival statistics of individual trials, see Supplementary Table 8. Source data are provided as a Source Data file.

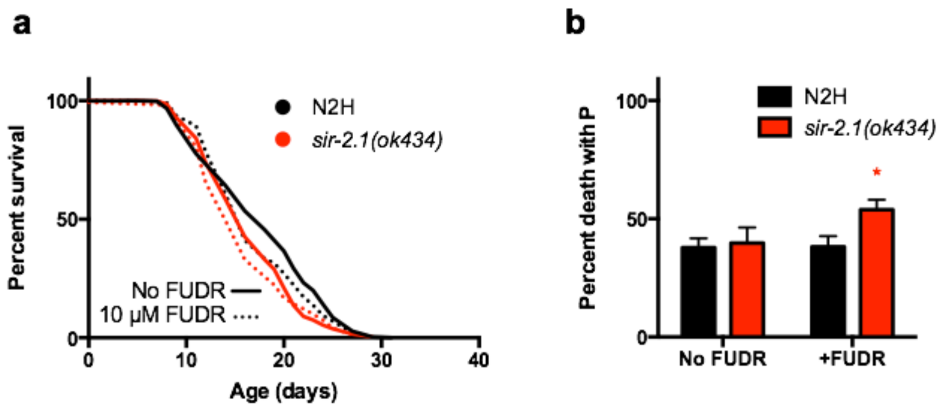

Supplementary Figure 8. The lifespan effect of *sir-2.1* null mutation.
(a) Lifespan and (b) P death frequency of outcrossed *sir-2.1(ok434)* mutant, in the presence and absence of FUDR. An increase in P death frequency caused by *sir-2.1* null mutation was only seen in FUDR-treated condition. See Supplementary Table 9 for full statistics. Source data are provided as a Source Data file.

Strain, treatment	Number of deaths/ censored	%P	All deaths			P deaths			p deaths		
			Mean lifespan (days)	% change vs. control	*p* vs. control (log rank)	Mean lifespan (days)	% change vs. control	*p* vs. control (log rank)	Mean lifespan (days)	% change vs. control	*p* vs. control (log rank)
N2H (control)	**[C] 335/25**	**36.6**	**18.7**			**12.8**			**22.6**		
	[1] 54/1	33.3	18.5			12.2			21.7		
	[2] 54/4	31.5	19.8			11.2			23.8		
	[3] 53/5	28.3	19.6			12.8			22.3		
	[4] 62/6	59.7	17.3			13.8			22.4		
	[5] 58/3	31.0	18.9			11.5			22.2		
	[6] 54/6	46.3	17.9			11.9			23.4		
N2M	**[C] 267/23**	**13.3**	**22.1**	**+16.6**	**<0.0001**	**13.6**	**+6.3**	**0.2260**	**23.5**	**+4.0**	**0.1721**
	[1] 60/0	8.3	21.6	+16.8	0.0105	11.6	-4.9	0.5944	22.5	+3.7	0.2945
	[2] 46/5	17.0	23.0	+16.2	0.0215	12.8	+14.3	0.0223	24.5	+2.9	0.4843
	[3] 58/4	13.8	22.6	+15.3	0.0089	14.0	+9.4	0.2366	24.0	+7.6	0.0450
	[4] 103/14	13.4	21.8	+26.0	0.0001	14.4	+4.3	0.1798	23.3	+4.0	0.2291
	[5] 58/4	15.5	21.0	+11.1	0.1114	11.6	+0.9	0.9206	22.8	+2.7	0.5692
	[6] 54/6	14.8	22.4	+25.1	0.0442	14.9	+24.8	0.0725	23.7	+1.3	0.6972
N2H + carbenicillin (control)	**[C] 169/22**	**0**	**27.7**								
	[1] 68/1	0	30.5								
	[4] 50/12	0	27.6								
	[6] 51/9	0	24.2								
N2M + carbenicillin	**[C] 157/35**	**0**	**28.5**	**+2.9**	**0.3766**						
	[1] 66/2	0	31.1	+2.0	0.6819						
	[4] 45/19	0	27.3	-1.1	0.9909						
	[6] 46/14	0	26.1	+7.7	0.1806						
N2H UV-treated OP50 (control)	**[C] 76/31**	**0**	**27.9**								
	[7] 37/16	0	27.6								
	[8] 39/15	0	28.3								
N2M UV-treated OP50	**[C] 66/49**	**0**	**27.5**	**-1.7**	**0.5672**						
	[7] 43/22	0	27.5	-0.4	0.6788						
	[8] 23/27	0	27.4	-3.2	0.5875						

Supplementary Table 1. Mortality deconvolution of N2H and N2M on proliferating and non-proliferating *E. coli*.

Trials were performed at 20˚C, with no FUDR. [C], combined data from all trials, [n], trial number. For statistics of P and p subpopulations, animals lost due to internal hatching or vulva rupture were excluded from statistical analysis rather than censored (see Methods). Sample sizes for P and p subpopulations can be estimated from total sample size and %P.

Strain	Number of deaths/ censored	%P	All deaths			P deaths			p deaths		
			Mean lifespan (days)	% change vs. control	*p* vs. control (log rank)	Mean lifespan (days)	% change vs. control	*p* vs. control (log rank)	Mean lifespan (days)	% change vs. control	*p* vs. control (log rank)
N2H (control)	**[C] 177/23**	**39.6**	**18.7**			**13.0**			**22.7**		
	[1] 62/6	59.7	17.3			13.8			22.4		
	[2] 58/4	34.5	18.7			12.6			21.9		
	[3] 57/13	28.1	20.2			11.8			23.5		
N2M	**[C] 218/18**	**13.8**	**22.2**	**+18.7**	**<0.0001**	**14.7**	**+13.1**	**0.0729**	**23.6**	**+4.0**	**0.3134**
	[1] 103/14	13.4	21.8	+26.0	0.0001	14.4	+4.3	0.6109	23.3	+4.0	0.2291
	[2] 57/2	14.0	22.4	+19.8	0.0148	12.6	+0.0	0.7200	24.0	+9.6	0.1754
	[3] 58/2	13.8	22.3	+10.4	0.0270	15.8	+33.5	0.0191	23.3	-0.9	0.3539
GA1947 *fln-2(syb202)*	**[C] 169/24**	**17.5**	**21.3**	**+13.9**	**0.0084**	**12.1**	**-6.9**	**0.1213**	**23.2**	**+2.2**	**0.6961**
	[1] 58/8	20.7	20.2	+16.8	0.0291	11.5	-16.7	0.0062	22.5	+0.4	0.7792
	[2] 52/6	17.3	21.7	+16.0	0.0489	11.3	-10.3	0.5251	23.9	+9.1	0.2406
	[3] 59/10	15.3	21.9	+12.3	0.1058	13.6	+15.3	0.1031	23.4	+4.0	0.4561

Supplementary Table 2. Mortality deconvolution of N2H-derived *fln-2(syb202)* Y800* mutant generated by CRISPR. Trials were performed at 20˚C, with no FUDR. [C], combined data from all trials, [n], trial number.

Strain	*fln-2* allele	Strain	*fln-2* allele	Strain	*fln-2* allele	Strain	*fln-2* allele
BC3559	*ot611*	FX311	+	RB754	*ot611*	VC172	*ot611*
BC4666	*ot611*	FX1146	+	RB925	*ot611*	VC199	*ot611*
BC5780	*ot611*	FX4687	+	RB1085	*ot611*	VC281	+
BC10060	*ot611*	FX5030	+	RB1206	*ot611*	VC426	*ot611*
BC10615	*ot611*	FX5539	+	RB1240	*ot611*	VC557	+
BC12677	*ot611*	FX6659	+	RB1288	*ot611*	VC754	*ot611*
BC16329	*ot611*					VC818	*ot611*
BC20063	*ot611*					VC1141	*ot611*
						VC1801	+
						VC3072	+

Supplementary Table 3. *fln-2* genotype of listed strains from *C. elegans* Expression Project (BC strains) and *C. elegans* Gene Knockout Project (FX, RB and VC strains).

Strain	Number of deaths/ censored	%P	All deaths			P deaths			p deaths		
			Mean lifespan (days)	% change vs. control	*p* vs. control (log rank)	Mean lifespan (days)	% change vs. control	*p* vs. control (log rank)	Mean lifespan (days)	% change vs. control	*p* vs. control (log rank)
GA707 *wuEx166[rol-6] fln-2(ot611)* (control)	**[C] 151/8**	**15.5**	**21.6**			**12.8**			**23.6**		
	[1] 50/6	24.5	20.0			12.1			23.8		
	[2] 49/1	12.2	22.5			15.7			23.4		
	[3] 52/1	9.6	22.2			11.8			23.3		
GA468 *geIs3[sir-2.1 rol-6] fln-2(ot611)*	**[C] 140/21**	**10.5**	**21.6**	**+0.0**	**0.9583**	**12.4**	**-3.1**	**0.4560**	**22.7**	**-3.8**	**0.0061**
	[1] 53/7	15.1	23.4	+16.6	0.0263	12.0	-0.8	0.7403	25.4	-6.3	0.1874
	[2] 44/7	6.8	20.6	-8.4	0.1043	12.7	-19.1	0.1066	21.2	-9.4	0.0583
	[3] 43/7	9.3	20.3	-8.5	0.1597	13.0	+10.2	0.6130	21.0	-13.6	0.1380
LG398 *geIs101[rol-6]* (control)	**[C] 177/11**	**45.1**	**18.3**			**12.2**			**23.8**		
	[1] 56/3	37.5	19.1			12.7			23.0		
	[2] 63/1	52.4	18.5			12.4			24.8		
	[3] 58/7	51.7	17.4			11.6			23.6		
LG394 *geIs3[sir-2.1 rol-6]*	**[C] 171/10**	**26.7**	**23.2**	**+26.8**	**<0.0001**	**13.9**	**+13.9**	**0.0045**	**26.7**	**+12.2**	**<0.0001**
	[1] 54/6	20.4	23.4	+22.5	0.0002	14.1	+11.0	0.0828	25.7	+11.7	0.0026
	[2] 58/3	31.0	24.5	+32.4	<0.0001	14.9	+20.2	0.0287	28.8	+16.1	0.0006
	[3] 59/1	30.5	21.8	+25.3	0.0005	12.8	+10.3	0.1932	25.7	+9.3	0.0161

Supplementary Table 4. Mortality deconvolution of the original *sir-2.1* overexpression strains.

Trials were performed at 20˚C, with 10 μM FUDR. [C], combined data from all trials, [n], trial number.

Strain	Number of deaths/ censored	%P	All deaths			P deaths			p deaths		
			Mean lifespan (days)	% change vs. control	*p* vs. control (log rank)	Mean lifespan (days)	% change vs. control	*p* vs. control (log rank)	Mean lifespan (days)	% change vs. control	*p* vs. control (log rank)
GA1907 *wuEx166 [rol-6]* (control)	**[C] 120/5**	**44.2**	**18.0**			**13.5**			**21.5**		
	[1] 62/3	43.5	18.2			13.0			22.3		
	[2] 58/2	44.8	17.7			13.9			20.7		
GA1909 *geIs3 [sir-2.1 rol-6]*	**[C] 112/18**	**29.3**	**21.5**	**+19.4**	**<0.0001**	**14.3**	**+5.9**	**0.2680**	**24.5**	**+14.0**	**0.0016**
	[1] 62/8	27.4	22.0	+20.9	0.0022	13.8	+6.2	0.5517	25.0	+12.1	0.0256
	[2] 50/10	32.0	20.9	+18.1	0.0024	14.8	+6.5	0.3319	23.8	+15.0	0.0126
GA1915 *geIs101 [rol-6]* (control)	**[C] 177/11**	**55.7**	**16.4**			**12.7**			**20.9**		
	[1] 60/2	50	17.2			12.0			22.4		
	[2] 55/4	63.6	16.0			13.0			21.3		
	[3] 62/5	51.6	15.9			13.1			19.0		
GA1913 *geIs3 [sir-2.1 rol-6]*	**[C] 153/27**	**25.4**	**21.8**	**+31.9**	**<0.0001**	**16.1**	**+26.8**	**<0.0001**	**23.7**	**+13.4**	**0.0002**
	[1] 51/10	25.5	22.4	+30.2	<0.0001	15.9	+32.5	0.0008	24.6	+9.8	0.0343
	[2] 46/13	26.1	21.4	+33.8	<0.0001	16.3	+25.4	0.0047	23.3	+9.4	0.1580
	[3] 56/4	25.0	21.5	+35.2	<0.0001	16.2	+23.7	0.0054	23.3	+22.6	0.0008

Supplementary Table 5. Mortality deconvolution of *sir-2.1* overexpression strains outcrossed with N2H.

Trials were performed at 20˚C, with 10 μM FUDR. [C], combined data from all trials, [n], trial number.

Strain	Number of deaths/ censored	%P	All deaths			P deaths			p deaths		
			**Mean lifespan (days)**	**% change vs. control**	***p* vs. control (log rank)**	**Mean lifespan (days)**	**% change vs. control**	***p* vs. control (log rank)**	**Mean lifespan (days)**	**% change vs. control**	***p* vs. control (log rank)**
GA707 *wuEx166 [rol-6] fln-2(ot611)* (control)	**[C] 87/23**<br>[1] 44/16<br>[2] 43/7	**11.4**<br>18.2<br>4.7	**22.8**<br>22.2<br>23.4			**12.8**<br>12.5<br>14.0			**24.1**<br>24.3<br>23.9		
GA468 *geIs3 [sir-2.1 rol-6] fln-2(ot611)*	**[C] 95/15**<br>[1] 53/7<br>[2] 42/8	**8.8**<br>15.1<br>2.4	**22.2**<br>23.4<br>20.8	**-2.6**<br>+5.4<br>-11.1	**0.4541**<br>0.2730<br>0.0135	**12.6**<br>12.0<br>17.0	**-1.6**<br>-4.0<br>+21.4	**0.5091**<br>0.7666<br>0.2253	**23.2**<br>25.4<br>20.9	**-3.7**<br>+4.5<br>-12.6	**0.3447**<br>0.4129<br>0.0117
LG398 *geIs101 [rol-6]* (control)	**[C] 110/11**<br>[1] 58/2<br>[2] 52/9	**32.4**<br>37.9<br>26.9	**18.3**<br>17.1<br>19.6			**12.4**<br>13.0<br>11.5			**21.1**<br>19.6<br>22.9		
LG394 *geIs3 [sir-2.1 rol-6]*	**[C] 105/16**<br>[1] 53/8<br>[2] 52/8	**30.8**<br>30.8<br>30.8	**19.4**<br>18.1<br>20.8	**+6.0**<br>+5.8<br>+6.1	**0.2507**<br>0.5077<br>0.3428	**13.2**<br>12.1<br>14.3	**+6.5**<br>-6.9<br>+24.3	**0.2702**<br>0.6665<br>0.0032	**22.2**<br>20.7<br>23.7	**+5.2**<br>+5.6<br>+4.9	**0.3429**<br>0.6979<br>0.2133

Supplemental Table 6. Mortality deconvolution of the original *sir-2.1* overexpression strains without FUDR. Trials were performed at 20˚C, with no FUDR. [C], combined data from all trials, [n], trial number.

Strain	Number of deaths/ censored	%P	All deaths			P deaths			p deaths		
			**Mean lifespan (days)**	**% change vs. control**	***p* vs. control (log rank)**	**Mean lifespan (days)**	**% change vs. control**	***p* vs. control (log rank)**	**Mean lifespan (days)**	**% change vs. control**	***p* vs. control (log rank)**
GA1907 *wuEx166 [rol-6]* (control)	**[C] 106/11**	**40.3**	**19.5**			**13.4**			**23.7**		
	[1] 51/7	33.3	21.1			13.2			25.0		
	[2] 55/4	47.3	18.0			13.5			22.1		
GA1909 *geIs3 [sir-2.1 rol-6]*	**[C] 105/11**	**42.8**	**17.8**	**-8.7**	**0.0122**	**13.0**	**-3.0**	**0.7444**	**21.6**	**-8.9**	**0.0054**
	[1] 51/5	39.2	19.4	-8.1	0.0273	13.9	+5.3	0.6353	22.9	-8.4	0.0121
	[2] 54/6	48.1	16.4	-8.9	0.1201	12.3	-8.9	0.2649	20.1	-9.0	0.1054
GA1915 *geIs101 [rol-6]* (control)	**[C] 160/14**	**51.6**	**16.1**			**12.4**			**20.2**		
	[1] 50/5	44.0	17.9			12.5			22.1		
	[2] 55/5	45.5	16.1			12.5			19.1		
	[3] 55/4	67.3	14.6			12.3			19.3		
GA1913 *geIs3 [sir-2.1 rol-6]*	**[C] 161/14**	**49.1**	**16.8**	**+4.3**	**0.2348**	**12.3**	-0.8	**0.5799**	**22.3**	**+10.4**	**0.1258**
	[1] 51/5	35.3	18.4	+2.8	0.7486	12.9	+3.2	0.6058	21.4	-3.2	0.7714
	[2] 55/5	52.7	16.3	+1.2	0.8403	11.7	-6.4	0.1425	21.5	+12.6	0.1513
	[3] 55/4	60.0	15.9	+8.9	0.1879	12.4	+10.2	0.9084	21.1	+15.2	0.2425

Supplementary Table 7. Mortality deconvolution of the outcrossed *sir-2.1* overexpression strains without FUDR. Trials were performed at 20˚C, with no FUDR. [C], combined data from all trials, [n], trial number.

Strain	Number of deaths/ censored	%P	All deaths			P deaths			p deaths		
			Mean lifespan (days)	% change vs. control	*p* vs. control (log rank)	Mean lifespan (days)	% change vs. control	*p* vs. control (log rank)	Mean lifespan (days)	% change vs. control	*p* vs. control (log rank)
NL3908 *pkIs1641[unc-119(+)] unc-119(ed3)III* (control)	**[C] 75/40**	**17.3**	**22.3**			**10.8**			**24.7**		
	[2] 41/19	22.0	21.2			10.4			24.3		
	[3] 34/21	11.8	23.6			11.5			25.2		
NL3909 *pkIs1642[unc-119(+) sir-2(+)] unc-119*	**[C] 74/42**	**24.3**	**22.9**	**+2.6**	**0.2375**	**11.8**	**+9.9**	**0.3654**	**26.4**	**+7.0**	**0.0454**
	[2] 44/16	25.0	22.2	+4.4	0.3239	11.5	+9.7	0.3896	25.8	+6.1	0.1777
	[3] 30/26	23.3	23.9	+1.4	0.3661	12.4	+8.1	0.6452	27.4	+8.8	0.0880
GA906 *pkIs1641(unc-119[+]) unc-119* (control)	**[C] 252/23**	**53.6**	**16.8**			**12.9**			**21.3**		
	[1] 52/8	55.8	16.4			13.5			20.1		
	[2] 53/7	52.8	16.0			12.6			19.9		
	[3] 46/8	52.2	18.1			12.6			24.0		
GA905 *pkIs1642[unc-119(+) sir-2(+)] unc-119*	**[C] 158/17**	**49.4**	**18.1**	**+7.6**	**0.0864**	**13.1**	**+1.5**	**0.7829**	**22.9**	**+7.7**	**0.1007**
	[1] 55/5	54.5	17.3	+5.3	0.6460	13.8	+2.4	0.6629	21.5	+6.7	0.9644
	[2] 59/1	47.5	17.3	+7.7	0.8766	12.7	+0.9	0.3152	21.4	+7.6	0.2997
	[3] 44/11	45.5	20.1	+11.2	0.1179	12.6	+0.1	0.8348	26.3	+9.5	0.0735
SCS004 *pkIs1641(unc-119[+]) unc-119* (control)	**[C] 154/21**	**54.5**	**17.2**			**12.9**			**22.3**		
	[1] 52/8	61.5	16.4			13.0			21.8		
	[2] 53/7	60.4	15.8			12.5			20.8		
	[3] 49/6	40.8	19.5			13.3			23.8		
SCS003 *pkIs1642[unc-119(+) sir-2(+)] unc-119*	**[C] 124/51**	**34.7**	**18.3**	**+6.6**	**0.2874**	**12.6**	**-2.3**	**0.6062**	**21.3**	**-4.4**	**0.1881**
	[1] 50/10	44.0	17.7	+8.4	0.4142	13.2	+1.6	0.7252	21.3	-2.2	0.5019
	[2] 29/31	44.8	17.1	+8.1	0.6066	12.2	-2.8	0.4883	21.1	+1.2	0.5880
	[3] 45/10	31.1	19.7	+1.0	0.8917	12.1	-8.4	0.3871	23.1	-2.9	0.5821

Strain, treatment	Number of deaths/ censored	%P	All deaths			P deaths			p deaths		
			Mean lifespan (days)	% change vs. control	*p* vs. control (log rank)	Mean lifespan (days)	% change vs. control	*p* vs. control (log rank)	Mean lifespan (days)	% change vs. control	*p* vs. control (log rank)
NL3908 *pkIs1641(unc-119[+]) unc-119(ed3)* + FUDR (control)	**[C] 89/30**	**26.7**	**23.1**			**15.0**			**26.0**		
	[2] 47/14	29.8	21.3			13.9			24.5		
	[3] 42/16	23.1	25.1			16.7			27.6		
NL3909 *pkIs1642[unc-119(+) sir-2(+)] unc-119* + FUDR	**[C] 114/7**	**24.6**	**30.3**	**+31.2**	**<0.0001**	**14.8**	**-1.2**	**0.6028**	**35.3**	**+35.7**	**<0.0001**
	[2] 58/5	24.1	27.3	+28.0	<0.0001	12.1	-12.8	0.1494	32.2	+31.2	<0.0001
	[3] 56/2	25.0	33.3	+32.6	<0.0001	17.6	+4.7	0.9828	38.6	+39.6	<0.0001
GA906 *pkIs1641(unc-119[+]) unc-119* + FUDR (control)	**[C] 155/21**	**56.8**	**16.8**			**13.5**			**21.2**		
	[1] 51/9	47.1	18.2			13.3			22.6		
	[2] 55/5	54.5	16.2			12.8			20.2		

	[3] 49/7	69.4	16.2			14.4			20.3		
GA905 *pkIs1642[unc-119(+) sir-2(+)] unc-119* + FUDR	**[C] 157/19**	**56.1**	**17.6**	**+4.4**	**0.3928**	**13.2**	**-2.5**	**0.5136**	**23.2**	**+8.5**	**0.1847**
	[1] 48/12	41.7	19.0	+4.5	0.9227	13.7	+3.4	0.4576	22.8	+1.0	0.4509
	[2] 58/2	63.8	16.4	+1.6	0.9275	12.4	-3.1	0.4438	23.6	+16.5	0.1643
	[3] 51/5	60.8	17.5	+8.2	0.2704	13.8	-4.0	0.4761	23.3	+14.7	0.1312
SCS004 *pkIs1641(unc-119[+]) unc-119* + FUDR (control)	**[C] 157/20**	**63.1**	**15.6**			**12.2**			**21.4**		
	[1] 55/5	65.5	15.3			11.4			22.5		
	[2] 57/3	54.4	14.7			11.1			19.1		
	[3] 45/12	71.1	17.2			14.3			24.3		
SCS003 *pkIs1642[unc-119(+) sir-2(+)] unc-119* + FUDR	**[C] 157/20**	**42.7**	**19.4**	**+24.4**	**<0.0001**	**13.5**	**+10.3**	**0.0020**	**23.8**	**+11.5**	**0.1246**
	[1] 47/13	40.4	19.7	+29.3	0.0059	13.7	+20.3	0.0001	23.8	+5.6	0.9540
	[2] 56/4	51.8	17.4	+18.0	0.0065	13.0	+17.2	0.0046	22.1	+15.9	0.0099
	[3] 54/3	35.2	21.3	+23.9	0.0227	14.1	-1.6	0.6717	25.2	+3.7	0.8052

Supplementary Table 8. Mortality deconvolution of original and outcrossed *pkIs1642 sir-2.1* overexpression strains with and without FUDR. Trials were performed at 20˚C, with 10 μM or no FUDR. [C], combined data from all trials, [n], trial number.

Strain, treatment	Number of deaths/ censored	%P	All death			P death			p death		
			Mean lifespan (days)	% change vs. control	*p* vs. control (log rank)	Mean lifespan (days)	% change vs. control	*p* vs. control (log rank)	Mean lifespan (days)	% change vs. control	*p* vs. control (log rank)
N2H (control)	**[C] 164/17**	**37.9**	**18.0**			**12.0**			**21.0**		
	[1] 54/6	31.5	17.9			9.8			21.1		
	[2] 58/3	31.0	18.9			11.5			22.2		
	[3] 52/8	42.3	17.0			13.8			19.3		
GA1934 *sir-2.1(ok434)*	**[C] 152/29**	**39.7**	**16.4**	**-8.8**	**0.0015**	**12.7**	**+5.8**	**0.1766**	**18.9**	**-10.0**	**<0.0001**
	[1] 49/11	34.7	16.2	-9.5	0.0104	12.6	+28.6	0.0021	18.2	-13.7	0.0001
	[2] 56/5	55.4	16.3	-13.8	0.0208	13.0	+13.0	0.0434	20.5	-7.7	0.1903
	[3] 47/13	29.8	16.0	-5.5	0.6623	12.0	-13.0	0.0829	18.5	-4.0	0.4606
N2H + FUDR (control)	**[C] 149/21**	**38.2**	**17.0**			**13.3**			**19.5**		
	[1] 39/11	48.7	17.1			12.9			21.1		
	[2] 50/10	30.0	18.0			13.8			19.8		
	[3] 60/0	41.7	16.2			13.2			18.3		
GA1934 *sir-2.1(ok434)* + FUDR	**[C] 159/14**	**53.8**	**15.4**	**-9.4**	**0.0425**	**12.5**	**-6.0**	**0.0933**	**18.7**	**-4.1**	**0.6881**
	[1] 49/3	42.9	15.5	-9.4	0.1462	12.0	-7.0	0.2310	18.1	-14.2	0.0319
	[2] 52/9	61.5	15.8	-12.2	0.2480	13.8	0.0	0.4648	19.1	-3.5	0.4270
	[3] 58/2	55.2	14.9	-8.0	0.4294	11.6	-12.1	0.0111	19.1	+4.4	0.4306

Supplementary Table 9. Mortality deconvolution of *sir-2.1(ok434)* mutant strains.

Trials were performed at 20˚C, with 10 μM or no FUDR. [C], combined data from all trials, [n], trial number.

Strain	Number of deaths/ censored	%P	All death			P death			p death		
			Mean lifespan (days)	% change vs. control	*p* vs. control (log rank)	Mean lifespan (days)	% change vs. control	*p* vs. control (log rank)	Mean lifespan (days)	% change vs. control	*p* vs. control (log rank)
DR1564 *daf-2(m41); fln-2(ot611)*	**[C] 112/31**	**6.0**	**28.1**			**13.1**			**29.1**		
(control)	[1] 60/13	8.3	25.1			14.4			26.1		
	[2] 52/18	3.8	31.5			10.0			32.3		
DR1547 *daf-2(m41); daf-12(m20)*	**[C] 92/36**	**54.9**	**21.4**	**-23.8**	**0.0069**	**11.4**	**-13.3**	**0.3396**	**33.9**	**+16.5**	**0.0322**
	[1] 49/15	53.1	20.8	-17.4	0.1186	12.3	-14.3	0.3382	30.2	+15.9	0.1054
	[2] 43/21	58.1	22.1	-29.6	0.0381	10.4	+4.0	0.6746	38.4	+18.9	0.0780
GA1945 *daf-2(m41)* (control)	**[C] 89/38**	**31.9**	**27.3**			**12.8**			**33.3**		
	[1] 42/26	35.7	23.0			14.9			27.6		
	[2] 47/12	27.7	31.1			9.8			37.6		
DR1547 *daf-2(m41); daf-12(m20)*	**[C] 92/36**	**54.9**	**21.4**	**-21.6**	**0.0005**	**11.4**	**-10.8**	**0.1488**	**33.9**	**+1.6**	**0.9812**
	[1] 49/15	53.1	20.8	-9.9	0.2326	12.3	-17.3	0.0452	30.2	+9.8	0.6143
	[2] 43/21	58.1	22.1	-28.9	0.0956	10.4	+5.9	0.4816	38.4	+2.1	0.5211

Supplementary Table 10. Mortality deconvolution of *daf-2* and *daf-2; daf-12* strains.

Animals were raised at 15˚C until L4, and shifted to 25ºC for lifespan analysis. No FUDR. [C], combined data from all trials, [n], trial number.

Strain	Number of deaths/ censored	%P	All death			P death			p death		
			Mean lifespan (days)	% change vs. control	*p* vs. control (log rank)	Mean lifespan (days)	% change vs. control	*p* vs. control (log rank)	Mean lifespan (days)	% change vs. control	*p* vs. control (log rank)
N2H (control)	**[C] 174/15**	**44.8**	**18.0**			**12.5**			**22.6**		
	[1] 62/6	59.7	17.3			13.8			22.4		
	[2] 58/3	31.0	18.9			11.5			22.2		
	[3] 54/6	22.0	17.9			11.4			23.4		
DA1116 *eat-2(ad1116)*	**[C] 152/25**	**14.3**	**22.9**	**+27.2**	**<0.0001**	**13.8**	**+10.4**	**0.0792**	**24.4**	**+8.0**	**0.0183**
	[1] 49/8	16.3	22.3	+28.9	0.0003	12.5	-9.4	0.4350	24.2	+8.0	0.2710
	[2] 54/6	11.1	23.9	+26.5	0.0001	15.2	+32.2	0.0170	25.0	+12.6	0.0050
	[3] 49/11	14.3	22.5	+25.7	0.0201	14.1	+23.7	0.1080	23.8	+1.7	0.7346
N2M (control)	**[C] 215/24**	**14.0**	**21.8**			**13.7**			**23.3**		
	[1] 103/14	13.4	21.8			14.4			23.3		
	[2] 58/4	15.5	21.0			11.6			22.8		
	[3] 54/6	14.8	22.4			14.9			23.7		
GA66 *eat-2(ad1116); fln-2(ot611)*	**[C] 141/40**	**9.4**	**23.7**	**+8.7**	**0.0043**	**16.2**	**+18.2**	**0.1444**	**24.5**	**+5.2**	**0.0149**
	[1] 44/17	13.6	24.1	+10.6	0.2197	15.0	+4.2	0.9793	25.5	+9.4	0.1552
	[2] 47/13	14.9	23.5	+11.9	0.0435	16.6	+43.1	0.0062	24.7	+8.3	0.0515
	[3] 50/10	4.0	23.9	+6.7	0.5114	18.5	+24.2	0.5083	24.1	+1.7	0.9300

Supplementary Table 11. Mortality deconvolution of *eat-2(ad1116)* outcrossed strains.

Trials were performed at 20˚C, with no FUDR. [C], combined data from all trials, [n], trial number.

Supplementary References

1 Viswanathan, M. & Guarente, L. Regulation of *Caenorhabditis elegans* lifespan by *sir-2.1* transgenes. *Nature* **477**, E1-2 (2011).

2 Burnett, C. *et al*. Absence of effects of Sir2 overexpression on lifespan in *C. elegans* and *Drosophila*. *Nature* **477**, 482-485 (2011).

3 Viswanathan, M., Kim, S. K., Berdichevsky, A. & Guarente, L. A role for SIR-2.1 regulation of ER stress response genes in determining *C. elegans* life span. *Dev. Cell* **9**, 605-615 (2005).

4 Ludewig, A. H. *et al*. Pheromone sensing regulates *Caenorhabditis elegans* lifespan and stress resistance via the deacetylase SIR-2.1. *Proc. Natl. Acad. Sci. U S A* **110**, 5522-5527 (2013).
