# Supplementary material for: A fln-2 mutation affects lethal pathology and lifespan in C. elegans
Source: Nat Commun. 2019 Nov 8;10:5087. doi: 10.1038/s41467-019-13062-z (PMC6841690; doi:10.1038/s41467-019-13062-z)
Supplement: Supplementary file 3 — Description of Additional Supplementary Files [file 41467_2019_13062_MOESM3_ESM.pdf]

Description of Additional Supplementary Files

File name: Supplementary Data 1

Description: Ziehm table with full mortality data

File name: Supplementary Movie 1

Description: 3-Dimensional SPIM image of an infected pharynx on day 8 of adulthood (20˚C) where the terminal bulb is infected with RFP labelled *E. coli*.
